# Characterization of Spirulina-Alginate Beads Formed Using Ionic Gelation

**DOI:** 10.1155/2019/7101279

**Published:** 2019-04-01

**Authors:** Deepak Rajmohan, Danielle Bellmer

**Affiliations:** ^1^Happy Family, 3380 W. Americana Terrace, Suite 360, Boise, ID 83706, USA; ^2^Biosystems & Agricultural Engineering, 108 Food and Agricultural Products Center, Oklahoma State University, Stillwater, OK 74078, USA

## Abstract

Spirulina (blue-green algae) is one of the cheapest sources of protein and essential vitamins. However, bitterness and bad flavor of spirulina protein may limit its use in food products. In this study, spirulina was encapsulated using ionic gelation to facilitate protein delivery. The objective was to study the effects of different types of gelation methods on particle size, texture, morphology, and crude protein content of the beads. Spirulina protein was encapsulated in alginate using both internal and external gelation methods and varying concentrations of sodium alginate and calcium chloride. A total of six different treatments were evaluated, and characterization of the beads included crude protein content, hardness measured using a texture analyzer, and thickness and width of the beads. The morphology was also studied using a scanning electron microscope (SEM). Results from the texture analysis show that the hardness of the external gelation beads is three times that of the internal gelation beads, and an increase in the alginate percentage in both gelation methods increased the firmness of the beads. The crude protein content was significantly higher with the beads formed using external gelation than with internal gelation. The SEM micrographs also show that the surface morphology of the beads produced with internal gelation has a more heterogeneous structure. Overall, the beads formed with external gelation were superior to those formed with internal gelation methods. Results from this study suggest that alginate is suitable for encapsulation of spirulina protein and these hydrogel beads could be used to enhance the protein delivery and facilitate the design of foods with alternative sources of protein.

## 1. Introduction

An increasing world population and depleting natural resources have created a need to develop a sustainable and cost-effective protein source. Today, protein malnutrition is a problem worldwide, and the World Health Organization reports that protein malnutrition is one of the largest contributors to child mortality [1]. Especially in developing countries, malnutrition is the cause of many health problems in young children, including increased risk of mortality, weakened immune system, and diminished cognitive capacity and school performance [2]. This situation has created a demand for an alternative source of protein that can replace the conventional and expensive plant or animal protein.

Microbial cells have the potential to provide an alternative source of protein around the world. The protein extracted from different microbial sources is known as “Single Cell Protein” (SCP). Primary sources of Single Cell Protein are bacteria, moulds, yeasts, and algae [3]. SCP has many advantages over animal and plant protein including that it is neither seasonal nor climate dependent [4]. Algae contain very high levels of complete protein, and they are also rich in lipids, minerals, vitamins, soluble fiber, and other bioactive compounds [5, 6]. Algae is considered to be a stable, traditional food for people in Mexico (*Spirulina platensis*) and for people in Chad (*Spirulina maxima*)[7].

Among algae proteins, spirulina is considered to be a powerhouse of nutrients and it is one of the cheapest sources of protein and essential vitamins [8]. It has high concentrations of beta carotene, vitamin B-12, iron, Gamma Linolenic Acid (GLA) and minerals. Spirulina is also one of the primary sources of natural phycocyanin, which is used as a natural color in food products like chewing gums, candies, dairy products, jellies, ice creams, soft drinks and used as a biochemical tracer in immunoassays [9]. The amino acid profile of spirulina is comparable with that of other conventional protein sources such as eggs; however, the microalgal protein may have lower biological value and net protein utilization than egg and casein [10]. Spirulina can also be more sustainably produced than other traditional protein sources. It requires 200 times less land and 50 times less water than beef to produce the same amount of protein [11].

Incorporation of bioactive proteins and peptides into food products is a challenging task since they are sensitive to chemical or biochemical degradation and susceptible to aggregation. These proteins also possess a potential to cause off-flavors like bitterness or astringency in the food products [12]. In general, algal proteins like chlorella and spirulina are marketed in the form of tablets and liquids. Several trial experiments have been conducted to add algal proteins to everyday food items like bread, pasta, and noodles, but incorporation of spirulina in food products resulted in a dark-green and a less acceptable “burnt” aftertaste [5, 6]. The unpleasant taste, bad flavor and dark-green color of spirulina limit its application in higher concentration. Not much research has been done involving the physicochemical modification of spirulina using processes such as emulsification, encapsulation, gelling, or bleaching [5].

Encapsulation using ionic gelation can be used to mask the bitter taste of spirulina. Ionic gelation is a chemical reaction between sodium alginate and calcium chloride, where the sodium ions are replaced by the calcium ions to form a gel-like structure. This unique property of the alginate makes it a suitable material for encapsulation of bioactive compounds and protein [12, 13]. The binding affinity of the alginate differs for various cations and is also dependent on the chemical composition of the alginate. Calcium is the most commonly used cation since it is nontoxic and inexpensive [14]. Alginate is also an excellent carrier material for protein delivery since proteins can be easily integrated into alginate-based formulations with mild conditions that minimize protein denaturation. Due to the inherent porosity and hydrophilic nature of the hydrogels, the release rate of protein from these gels is instant [15].

Alginates can be cross-linked by external or internal gelation methods. In the external gelation or diffusion method, the cations diffuse from the external medium into the interior of an alginate phase to form the hydrogel beads. The bioactive compound to be encapsulated is mixed with the alginate solution, and then the solution is extruded dropwise into an aqueous solution with cross-linking cations (calcium chloride solution) to form gelation [14]. For internal gelation, the cations are released from the interior of the alginate phase to form the hydrogel beads. The bioactive substance is mixed with the solution of cations and dropped into an alginate solution; the cation is released by acidification of the medium [16].

In this study, spirulina-alginate beads were prepared using both external and internal gelation methods with the extrusion technique. The effects of sodium alginate concentration, calcium chloride concentration and the type of gelation on the protein retention, morphological characteristics, and size and texture of the beads were studied. The goal was to identify the most suitable formulation of spirulina-alginate beads to be incorporated into a food product.

## 2. Materials and Methods

### 2.1. Materials

Spirulina powder (Spirulina platensis, ID: 7199) was purchased from Nuts.com, NJ, USA. Sodium alginate (W201502) was purchased from Sigma- Aldrich, USA and calcium chloride was purchased from Modernist Pantry, York, ME, USA. Sodium dodecylbenzene sulfonate (289957) was purchased from Sigma- Aldrich, USA, and Polysorbate 80/ Sorbitan monooleate was purchased from Vantage, Gurnee, IL, USA. Maltodextrin (DE = 18) was purchased from Myprotein, USA.

### 2.2. Preparation of Spirulina-Alginate Beads by External Gelation

**Figure 1:**
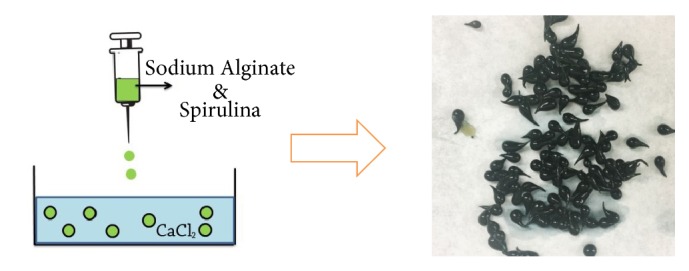
Bead formation using the external gelation process.

For preparation of beads using the external gelation process, the plain alginate solution was prepared by dissolving sodium alginate (1% w/w or 7% w/w) and Polysorbate-80 surfactant (1%w/w) in distilled water stirring at 65°C for 20 minutes. The spirulina powder was then mixed with the previously prepared alginate solution to obtain 15 % w/w concentration at 65°C for 20 minutes until it formed a homogeneous solution. The cross-linking solution (10% or 15% w/w) was prepared by dissolving calcium chloride powder in distilled water. The spirulina-alginate solution was drawn into a 3ml syringe with a 26 G needle and dropped manually into the cross-linking solution to form the alginate beads. As shown in Figure 1, the spirulina-alginate solution was extruded into the calcium chloride solution to form small teardrop shaped spirulina-alginate hydrogel beads. The manual extrusion process was slowed down to form beads of uniform size and shape. The beads were then filtered using a strainer, rinsed with distilled water, and stored under refrigeration until further analysis or use.

### 2.3. Preparation of Spirulina-Alginate Beads by Internal Gelation

For the internal gelation process, the plain alginate solution was prepared by dissolving sodium alginate (0.5% w/w or 1.5% w/w) and SDS (0.5% w/w) in distilled water stirring at 65°C for 20 minutes. The cross-linking solution was prepared by dissolving calcium chloride (2 % w/w) in distilled water. Maltodextrin (10% w/w) was used to adjust the viscosity of calcium chloride solution and ensure that the alginate beads were of uniform shape. Spirulina powder was mixed with calcium chloride solution to reach 15% w/w concentration. The spirulina-calcium chloride solution was drawn into a 3ml syringe with a 26 G needle and dropped manually into the alginate solution to form the beads. The formed beads were filtered using a strainer, rinsed using distilled water, and stored under refrigeration.

### 2.4. Experimental Treatments

Preliminary trials were conducted to examine the effect of the concentrations of sodium alginate and calcium chloride on gel formation. More than 30 different formulations were developed using different concentrations of sodium alginate and calcium chloride. All preliminary formulations were evaluated for crude protein and hardness of the beads. With external gelation, the beads were not formed when the concentration of calcium chloride was below 10% or the concentration of sodium alginate was below 1%. With internal gelation, the beads were not formed when the concentration of calcium chloride was above 2%. The range of concentrations of sodium alginate and calcium chloride selected for internal and external gelation were determined from the preliminary trials. A total of six different treatments were evaluated, including the following:

- External gelation: 1% sodium alginate and 10% calcium chloride
- External gelation: 1% sodium alginate and 15% calcium chloride
- External gelation: 7% sodium alginate and 10% calcium chloride
- External gelation: 7% sodium alginate and 15% calcium chloride
- Internal gelation: 0.50% sodium alginate and 2% calcium chloride
- Internal gelation: 1.5% sodium alginate and 2% calcium chloride

### 2.5. Determination of Size/Dimension of Beads

Samples of 10 spirulina-alginate beads obtained from each formulation and type of gelation were taken at random and measured with a digital caliper (ROHS CE Digital Caliper–SH20, China) to measure their width and length.

### 2.6. Scanning Electron Microscope: Morphological Studies

A pair of beads from each formulation and type of gelation method was viewed under a scanning electron microscope (Joel JSM 6360, Peabody) to determine both external and cross-sectional morphology. The beads were attached to stubs using adhesive tape and coated with gold. Finally, the beads were examined using an acceleration voltage of 10 kV at 25x and 50x magnification.

### 2.7. Determination of Textural/Mechanical Properties

The texture of the spirulina-alginate bead was analyzed using a texture analyzer (TA-XT 2i), and the compression testing was performed using a cylindrical probe. The samples were examined at a test speed of 0.5 mm/s, over a varied distance adjusted based on the dimensions of the samples in order to achieve complete compression. The maximum force (N) needed for compression represents the maximum resistance of the bead to compression of the probe, which indirectly gives an indication of the hardness of the samples. In order to obtain representative results of the hardness of the beads, experiments were performed in triplicate (with ten samples per experiment) and expressed as mean ± SD

### 2.8. Protein Analysis

The Dumas method (AOCS Official Method Ba 4e-93) for estimation of crude protein is based on combustion of the whole sample in an oxygen- enriched environment at 950°C in order to ensure complete combustion. All samples were analyzed for crude protein content using the Dumas method in triplicate. Samples (10 g) from each formulation were dried at 102°C for 18 hours and homogenized. The homogenized samples were analyzed for percent protein using a Leco combustion instrument (TruSpec N -630, St. Joseph, MI).

### 2.9. Encapsulation Efficiency

The encapsulation efficiency (%) of the alginate beads was determined by dividing the amount of spirulina remaining in the beads by the initial amount of spirulina added to each formulation. The amount of spirulina remaining in each formulation was determined based on the protein content of the beads and the total protein content of spirulina.

### 2.10. Statistical Analysis

The research study was designed as a completely randomized design. The ANOVA procedure was used to evaluate any significant differences between the gelation methods in terms of bead dimensions, protein content, and hardness of the beads. A generalized linear model was used with different factors being the dependent variables and treatments being the independent variables. Tukey's Studentized Range Test was used to detect the significantly different treatments using *α* =0.05.  Table 1 shows the sample size for each dependent variable.

## 3. Results and Discussion

### 3.1. Size and Dimension of Spirulina-Alginate Beads

Spirulina beads were prepared using both external and internal gelation methods, and varying levels of sodium alginate and calcium chloride. Differences in the formation methods resulted in different size beads. Due to the formation method, the bead shapes were not truly spherical but were more teardrop shaped, so two different dimensions were measured, termed thickness and length.  Table 2 shows the mean thickness (mm) and length (mm) of the beads for each formulation and gelation mechanism. The thickness and length measurements were analyzed by ANOVA. The external gelation (EG) beads had a mean thickness around 2 mm, whereas internal gelation (IG) beads had a mean thickness around 1.5 mm. Mean length of the external gelation beads ranged between 2.11 mm and 4.5 mm, and the mean length of the beads formed with internal gelation was approximately 3 mm. Irrespective of the gelation method, an increase in the concentration of sodium alginate significantly increased the thickness of the beads. This finding can be attributed to a less cross-linked gel, which consequently decreases syneresis. Syneresis is defined as a release of water from the gel with a consequent decrease in its dimensions [17]. However, an increase in calcium chloride concentration, while keeping the alginate concentration constant, did not significantly affect either thickness or length of the beads.

For incorporation into food products, the smallest possible beads would be ideal, because they are easier to ‘hide' in existing food products [18]. Bead size in these experiments was controlled by the diameter of the syringe needle used to prepare the beads. Obviously, a smaller diameter needle will create smaller beads. However, the limiting factor in this case was the pressure required to dispense the droplets, which was conducted by hand. In a commercial setting, it is likely that an extruder would be used to generate the beads, and therefore, much higher pressures and smaller outlet diameters would be possible.

### 3.2. Scanning Electron Microscope (SEM): Morphological Studies

**Figure 2:**
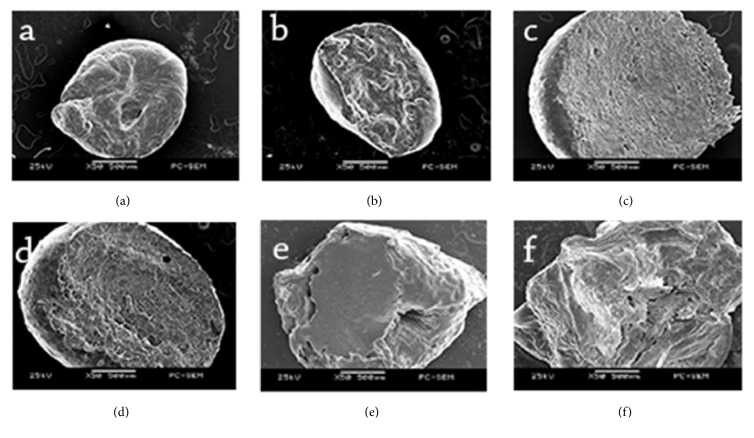
SEM micrographs showing the cross-sectional morphology of spirulina-alginate beads at 50x magnification made with (a) EG: alginate 1%, CaCl_2_ 10%, (b) EG: alginate 1%, CaCl_2_ 15%, (c) EG: alginate 7%, CaCl_2_ 10%, (d) EG: alginate 7%, CaCl_2_ 15%, (e) IG: alginate 0.5%, CaCl_2_ 2%, and (f) IG: alginate 1.5%, CaCl_2_ 2%.

A scanning electron microscope was used to evaluate the structural differences among the spirulina beads prepared in different ways. The internal structures of the beads prepared for each of six treatments are shown in Figures 2(a)–2(f). The SEM micrographs reveal differences in the cross-sectional morphology of external gelation beads and internal gelation beads. Beads obtained by external gelation show a more smooth and rigid exterior (Figures 2(a), 2(b), 2(c), and 2(d)), whereas beads formulated by internal gelation show a soft and heterogeneous exterior (Figures 2(e) and 2(f)). This phenomenon has been observed in other studies as well [19, 20]. The structure obtained by external gelation can be attributed to the formation of the gel layer on the surface of the droplet, which yields a rigid exterior [16, 21]. The calcium ions would first cross-link with the bead surface, which would draw the polymer chains closer to form a less permeable surface to the diffusion of calcium ions into the interior. This phenomenon results in a highly cross-linked surface and less cross-linked core [21]. This behavior is in accordance with the results reported by Aceval Arriola et al. [13] during the encapsulation of aqueous leaf extract of stevia rebaudiana. The external gelation beads appeared to have a more porous interior than the internal gelation beads due to the inward movement of Ca^2+^ ions from the shell to the core (Figures 2(a), 2(b), 2(c), and 2(d)). In contrast, with internal gelation, diffusion of calcium ions from the core to the surface leads to a more homogeneous internal structure. A similar structure was observed by Lupo et al. [16] during encapsulation of cocoa extract by both internal and external gelation methods.

### 3.3. Textural/Mechanical Properties

**Figure 3:**
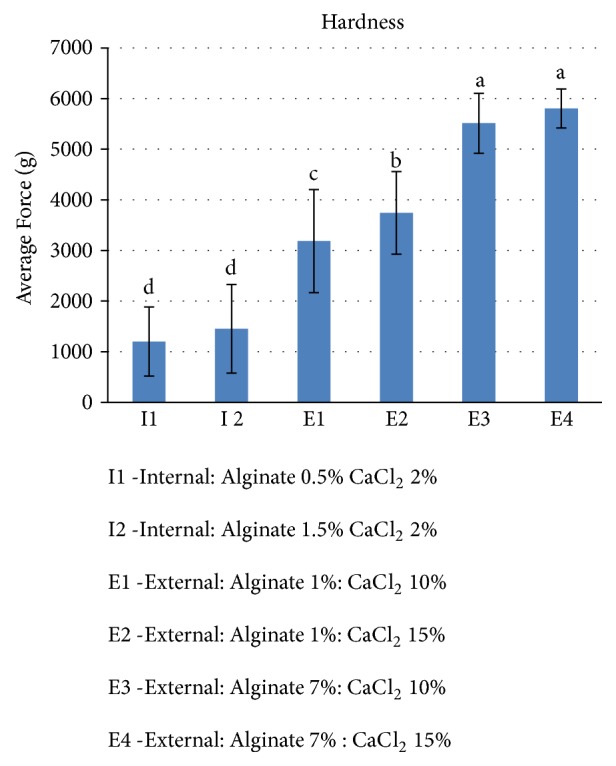
Hardness of spirulina-alginate beads for different formulations. Error bars represent ±S.D (n = 30). The bars with different letters are significantly different (*α* =0.05).

A texture analyzer was used to evaluate the hardness of the beads. A compression test was used to determine the maximum force required for complete compression of the spirulina – alginate beads, which indicates the hardness of the beads. The hardness data were analyzed by ANOVA.  Figure 3 shows the average hardness for each of the six different bead preparation treatments. The external gelation beads with 7% alginate had a maximum force of around 5600 g, but the external gelation beads with 1 % alginate had a maximum force of around 3500g. In the case of internal gelation, beads had a maximum force of around 1300g.

The hardness of the beads prepared by external gelation was significantly higher than the hardness of the beads prepared by internal gelation. This behavior is in agreement with the results reported by Lupo et al. [16] for the encapsulation of cocoa extract. The concentration of calcium chloride did not significantly influence the hardness of the beads formulated by internal gelation. However, the increase in calcium chloride concentration increased the hardness of the beads formed by external gelation with 1 % alginate from an average of 3186 g to 3744 g. It can also be seen that irrespective of the gelation methodology, beads with higher alginate concentration were harder than the beads with lower alginate concentration, which was expected.

Overall, the spirulina beads produced using external gelation with 7% alginate and 15% calcium chloride had the maximum resistance against compression and exhibited the greatest hardness. Alginate beads are largely used for food applications, and therefore they should possess suitable mechanical properties to withstand the stresses exerted during food processing [17].

### 3.4. Protein Analysis

**Figure 4:**
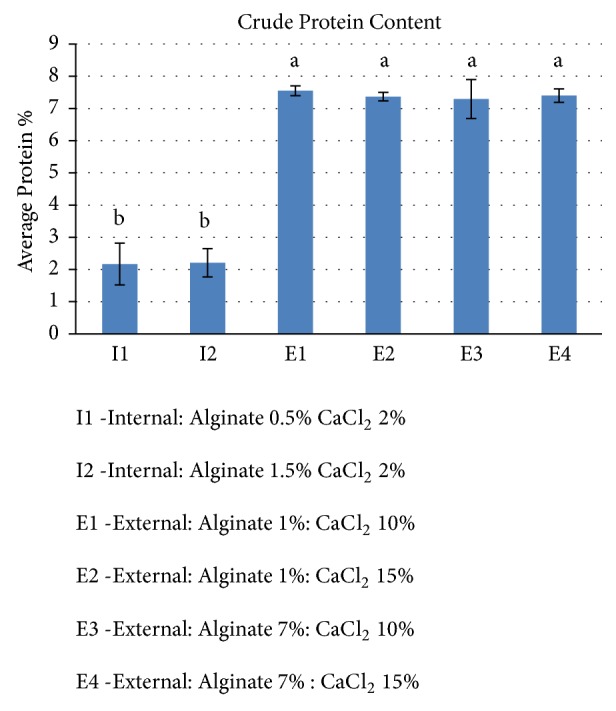
Crude protein content of spirulina-alginate beads for different formulations. Error bars represent ±S.D (n= 3). The bars with different letters are significantly different (*α* =0.05).

The protein content of the beads produced using each of the six different treatment methods was evaluated using the Dumas method. The data showing crude protein of the spirulina- alginate beads was analyzed by ANOVA.  Figure 4 shows the crude protein content of spirulina-alginate beads prepared by external gelation and internal gelation. From the figure, it can be seen that external gelation beads possess higher protein content than the internal gelation beads. The external gelation beads had protein content ranging between 7.29% and 7.59%, while internal gelation beads had protein content around 2.2%. The method of gelation had a significant impact on the protein content of the beads. However, in both external and internal gelation, the concentration of sodium alginate or calcium chloride did not significantly influence the protein content of the formulated beads.

### 3.5. Encapsulation Efficiency

The encapsulation efficiency was determined based on the fraction of protein in the initial mixtures before forming beads compared to the amount of protein in the final beads. From the protein content graph (Figure 4), it is clear that gelation method has a huge influence on the amount of protein retained, which directly correlates to the encapsulation efficiency. Irrespective of the sodium alginate and calcium chloride concentration, external gelation beads had an encapsulation efficiency around 78%, and internal gelation beads had an encapsulation efficiency around 23%. Overall, the encapsulation efficiency of external gelation beads was significantly higher than the encapsulation efficiency of internal gelation beads.

## 4. Conclusions

This study showed that it is possible to encapsulate spirulina protein using ionic gelation. Irrespective of the gelation method, an increase in the concentration of sodium alginate significantly increased the thickness of the beads. However, an increase in calcium chloride concentration did not significantly affect either thickness (mm) or length (mm) of the beads. External gelation beads exhibited a more uniform, homogeneous morphology compared to internal gelation beads. The hardness of the beads prepared by external gelation was significantly higher than the hardness of the beads prepared by internal gelation. The external gelation beads also possessed significantly higher protein content than the internal gelation beads and therefore exhibited much higher encapsulation efficiency than the internal gelation beads. Findings from this study suggest that alginate is suitable for encapsulation of spirulina protein and in the future these beads could be used for incorporation of alternative protein sources into food products.

## Figures and Tables

**Table 1:** Dependent variables and number of observations for statistical analysis.

Dependent Variable	Number of Observations (n)
Thickness (mm)	60 (6 treatments *∗* 10 reps)
Length (mm)	60 (6 treatments *∗* 10 reps)
Hardness (g)	180 (6 treatments *∗* 30 reps)
Protein Content (%)	18 (6 treatments *∗* 3 reps)

**Table 2:** Mean length and thickness of spirulina-alginate beads.

Gelation	Sodium Alginate %	Calcium Chloride %	Thickness (mm)	Length (mm)
External	1%	10%	1.40 ± 0.09c	2.11 ± 0.25c
	1%	15%	1.58 ± 0.09cb	2.08 ± 0.16c
	7%	10%	2.48 ± 0.09a	4.77 ± 0.30a
	7%	15%	2.39 ± 0.017a	4.48 ± 0.25a
Internal	0.50%	2%	1.42 ± 0.12c	3.03 ± 0.23b
	1.50%	2%	1.68 ± 0.23b	2.98 ± 0.28b

Data reported is mean ± standard deviation (n=10). Values for each treatment with different letters are significantly different (*α* = 0.05).

## Data Availability

The data used to support the findings of this study are available from the corresponding author upon request.
